# The Papua New Guinea medical supply system - documenting opportunities and challenges to meet the Millennium Development Goals

**DOI:** 10.1186/2052-3211-7-5

**Published:** 2014-05-19

**Authors:** Andrew N Brown, Ben Gilbert

**Affiliations:** 1 People that Deliver, UNICEF Supply Division, Copenhagen, Denmark; 2 Discipline of Pharmacy, Faculty of Health, University of Canberra, Canberra, ACT, Australia

**Keywords:** Essential medicines supply, Supply chain management, Pharmacy, Papua New Guinea, Competency, Sustainable health systems, Interview, Focus group, Workplace observation survey, Millenium development goals

## Abstract

**Objectives:**

Limited human resources are widely recognised as an impediment to achieving the health-related Millennium Development Goals in Pacific Island Countries, with the availability of medical supplies and suitably trained health personnel crucial to ensuring a well-functioning medical supply chain. This paper presents our findings as we seek to answer the research question ‘What factors influence the availability of medical supplies within the health facilities of Papua New Guinea?’

**Methods:**

We used a qualitative, triangulated strategy using semi-structured interviews, workplace observation and semi-structured focus groups. The parallel use of the interview tool and workplace observation tool allowed identification of ‘know-do’ gaps between what the interviewee said they did in their work practices, and the actual evidence of these practices. Focus groups provided further opportunities for raising and elaborating issues.

**Results:**

During 2 weeks of data collection we conducted 17 interviews and 15 observational workplace surveys in 15 facilities. Sixteen health personnel participated in 3 focus groups across 2 provinces and one district. An array of medical supply issues across all levels of the medical supply chain were revealed, including standard operating procedures, facilities, transport, emergency medical kits, the cold chain and record keeping. The influence of health worker training and competency was found to be common across all of these issues.

**Conclusion:**

The factors influencing the availability of medical supplies in PNG consist of a range of interrelating issues, consisting of both simple and complex problems involving the different levels and cadres of workers within the medical supply chain. Health systems sustainability theory suggests that a coordinated approach which addresses the inter-related nature of these issues, led by the PNG government and supported by suitable development partners, will be required for sustainable health systems change to occur. These changes are necessary for PNG to meet the health-related Millennium Development Goals.

## Introduction

We have previously presented the link between limited human resources for medical supply chains and the ability to achieve the health-related Millennium Development Goals (MDGs), through a Vanuatu case study [1]. In that article we emphasised that much maternal and child morbidity and mortality may be prevented by having ready access to essential medical supplies provided by appropriately trained health personnel (where medical supplies include medicines and medical sundries e.g. syringes and dressings). The links between the MDGs and medical supply chains are described in Table 1[1].

**Table 1:** **The relationship between the Millennium Development Goals (MDGs) and medical supply chains**[1]

MDG 4. Reduce child mortality	Pneumonia, diarrhoea, malaria and AIDS account for 43 per cent of all deaths in under-fives with most of these lives saved through low-cost prevention and treatment measures including antibiotics for acute respiratory infections, oral rehydration for diarrhoea, and immunisation.
MDG 5. Improve maternal health	More than 80 per cent of maternal deaths are caused by conditions such as haemorrhage, sepsis, and hypertensive diseases of pregnancy, each requiring medication and the use of medical sundries. It is estimated that meeting the unmet needs for contraception alone could cut, by almost a third, the number of maternal deaths.
MDG 6. Combat HIV/AIDS, malaria, and other diseases	Each disease has medication as part of the treatment protocol.
MDG 8. Develop a global partnership for development	In cooperation with pharmaceutical companies, provide access to affordable essential medicines in developing countries.

As in other Pacific Island Countries (PICs), the availability of medical supplies and suitable personnel in adequate numbers with appropriate competency is crucial to ensure a well-functioning medical supply chain in Papua New Guinea (PNG) [2].

The research presented in this article provides further country-based detail supporting the International Pharmaceutical Federation (FIP) [3], World Health Organisation (WHO) [4] and the former Australian Agency for International Development (AusAID) [5] observations that insufficient numbers and inadequate competency of pharmacy personnel is a contributor to continued problems in maintaining reliable medical supplies chains.

The United Nations Population Fund (UNFPA) and the University of Canberra (UC) are working together to investigate the competencies required by personnel to operate medical supply chains, and developing appropriate and sustainable approaches to health personnel competency development [6].

The geographical and cultural context of PNG is an important influence on supply chain management issues. PNG is located immediately north of Australia in the South Pacific Ocean, occupying the eastern half of the island of New Guinea which it shares with Indonesia. PNG covers an area of approximately 463,000 km^2^ and a population of about 6.5 million [7]. It is the largest and most populated PIC. The country is divided into 4 regions with 18 provinces, one autonomous region and one district (Highlands Region, Islands Region, Momase Region, Papua Region, autonomous region of Bougainville and the National Capital District) [7].

Provincial allegiances and organisation influences many aspects of PNG life, including provision of government services and politics. English, the pidgin Tok Pisin, and Hiri Motu are the official languages of PNG; however there are nearly 900 indigenous languages. 80% of the population resides in rural areas [8].

Communicable diseases, including pneumonia, malaria, tuberculosis, diarrhoea, meningitis and, increasingly HIV/AIDS, remain the leading causes of morbidity and account for around 50% of mortality [9]. The incidence of non-communicable diseases is rising, creating the double burden observed in most developing countries [9].

Within the public sector, management responsibility for hospitals and rural health services within provinces is divided [10]. The PNG Ministry of Health (MoH) manages the provincial hospitals, while provincial and local governments are responsible for all other services (health centres, rural hospitals and aid posts), known collectively as rural health services [10].

Health services are provided by government and church providers (which are both financed primarily from public sector funds), enterprise-based services (e.g. the mines), a small private sector, and traditional healers. A tiered structure of Area Medical Stores (AMSs), provincial transit stores, provincial hospitals and rural health services provides the basis of the medical supply chain, using a ‘pull’ or demand system coordinated by the Medical Supply Branch (MSB) of the MoH [10].

**Figure 1:**
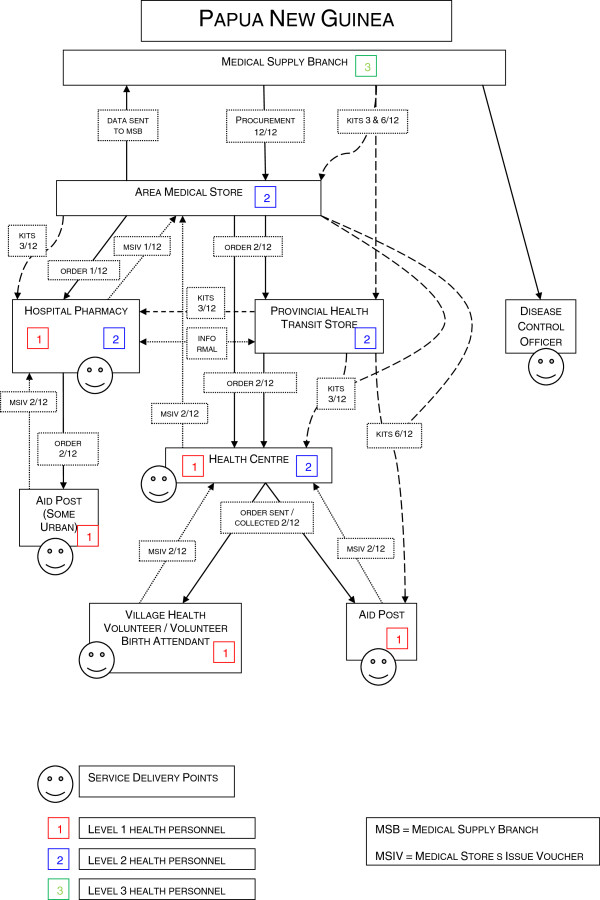
Schematic representation of the Papua New Guinea medical supply chain.

MSB is the administrative arm of the medical supply chain, responsible for procuring and distributing medical supplies to AMSs and provincial transit stores. There are six AMSs in PNG receiving stock either directly from international ports or transferred from the major AMSs of Badili (Port Moresby) and Lae. They distribute medical supplies directly to service delivery points or to provincial transit stores. Provincial transit stores are not medical facilities, but act as staging posts for medical supplies received from AMSs and are responsible for distributing to service delivery points. Provincial hospital pharmacies receive medical supplies from AMSs or provincial transit stores, dispense medicines to outpatients, and supply hospital wards, while rural health services receive supplies from provincial transit stores or an AMS. Figure 1 provides a schematic diagram of the PNG medical supply chain. We have used a Level nomenclature to broadly identify the medical supply responsibilities of workers at each facility:

• Level 3 workers are responsible for procuring medical supplies at the country level, and distribute supplies to Level 2 and Level 1 workers. They may typically be senior pharmacists, administrators, health program managers or other senior bureaucrats.

• Level 2 workers receive supplies from Level 3 workers, and distribute supplies to Level 1 workers, and also to patients if they are health professionals. They may typically be nurses, hospital pharmacists or health program coordinators. Some have a sole logistics role with no health qualification (e.g.: transit store manager).

• Level 1 workers receive supplies from Level 3 and Level 2 workers and distribute supplies to patients. They may typically be nurses or volunteer health workers with on-the-job training.

In addition to the ‘pull’ ordering system, a parallel ‘push’ system of emergency medical supply kits is managed by MSB, with standardised kits of medical supplies distributed up to four times a year to all health facilities in the country. This was originally intended to be an emergency response to a time several years before this study when PNG experienced significant medical supply chain failure; however it was observed to be continuing despite the original emergency passing.

### Research aim

The aim of this study was to determine the factors which influence the availability of medical supplies within the health facilities in PNG, with a further objective to document the medical supply activities undertaken within the various facilities and cadres of the PNG medical supply chain.

The results of this study will further inform the development of strategies to improve the availability of medical supplies in PNG specifically and PICs in general, and inform our understanding of the competencies required by healthcare personnel to complete this aspect of their work.

## Methods

To answer the research question we used a qualitative, triangulated strategy using semi-structured interviews, workplace observation surveys and semi-structured focus groups. We conducted these during 2 weeks of in-country fieldwork, with the assistance of PNG and provincial UNFPA counterparts and other provincial and facility staff. All interviews and focus groups were audio recorded.

We visited 3 areas, comprising 1 island province, 1 urban mainland district and 1 rural mainland province. Choice of areas was primarily based upon geography and accessibility during the study period and availability of area staff to guide us. Within each area we selected service delivery points to include examples each of AMSs, transit stores, hospitals and health centres. The facilities were chosen to allow observation of several complete examples of the medical supply chain from the AMS to moderately remote service delivery points, passing through all levels of the supply chain.

We developed the interview tool and workplace observation tool from existing WHO medicines supply indicator survey concepts [11], informed by the Medication Safety Self-Assessment for Australian Hospitals [12]. These sources were adapted to the context of PICs based upon our previous individual experiences living, working and conducting medicines supply consultancy and research activities in PICs. This adaptation focussed on collecting useful data about our observation and measurement of the application of work practices.

During fieldwork, one of us alternately administered the interview to the identified senior medical supply staff at each facility, whilst the other completed the workplace observation tool, sometimes with assistance from PNG and provincial UNFPA counterparts and other provincial and facility staff. We compared our findings before leaving the facility and evidence of practice was actively sought to reduce the risk of inadvertent omission. We collected qualitative data exploring technical and behavioural aspects of work practices, and quantitative demographic data about workers and facilities.

The parallel use of the interview tool and workplace observation tool allowed us to identify ‘know-do’ gaps between what the interviewee said they did in their work practices, and the actual evidence of these practices.

To complete the triangulation of data, allow elaboration of identified issues, and to ensure key issues were not overlooked, we developed a semi-structured focus group using key elements of the ‘World Café’ methodology [13]. Focus groups were conducted in each of the three areas after facility visits were completed, with workers from the previously studied facilities and other area facilities invited to attend.

We have previously used this approach to focus group facilitation in several different cultures to reduce hierarchical sensitivities in mixed cadre environments. It has been seen to be successful in empowering individuals to contribute as well as encouraging them to consider the opinions of others in a relaxed environment [1,13].

We also identified and clarified further issues in an informal way during the significant amount of time spent travelling with the PNG and provincial UNFPA counterparts and other provincial staff. Due consideration was given to any potential bias in issues raised in this way, and we actively sought independent supporting evidence.

We anonymised our collected data and pooled and organised it using the web-based survey application ‘Survey Monkey’. We then performed a manual thematic analysis, prioritising recurring issues and cross referencing them for validity across the three data collection methods. Issues were prioritised based on the frequency of their appearance across the data sources and the consistency of the information received. For example, issues which were raised by many people and which were expressed in consistent ways were considered to be more important and relevant. Issues which had practical connections to other issues were then collated into themes. Audio recordings were replayed in case of doubt.

We did not perform quantitative analysis of the qualitative data as the small sample size did not justify it, and thematic analysis is considered a suitable method to achieve the purposes of this research.

This methodology, and the tools used, was approved by the Human Ethics Committee of the University of Canberra (project number 10–85), and has been previously used and validated in Vanuatu [1].

## Results

During 2 weeks of data collection (8-21st August 2010) we conducted 17 interviews and 15 observational workplace surveys in 15 facilities. Sixteen health personnel participated in 3 focus groups across the 3 areas.

### Participant and facility demographics

The interviewees had a wide range of primary professional groupings, with nursing (25% n = 4) and pharmacy staff (44% n = 7) being the most common. Three worked in urban locations, 8 in provincial facilities and 6 in rural/remote facilities. They had worked at their current facility an average of 11.5 years (range 3- > 20 years), and in their current health professional capacity for 16 years on average (range 5–34 years). Interviewees worked at 15 different facilities comprising AMSs x 2, the national hospital x 1, provincial hospitals x 2, district hospital x 1, provincial transit stores x 2, government x 5, and church run health centres x 2. The range of facilities visited and personnel interviewed provides a suitably representative view of the issues around medical supply in the areas visited, which may be cautiously extrapolated to PNG as a whole.

**Figure 2:**
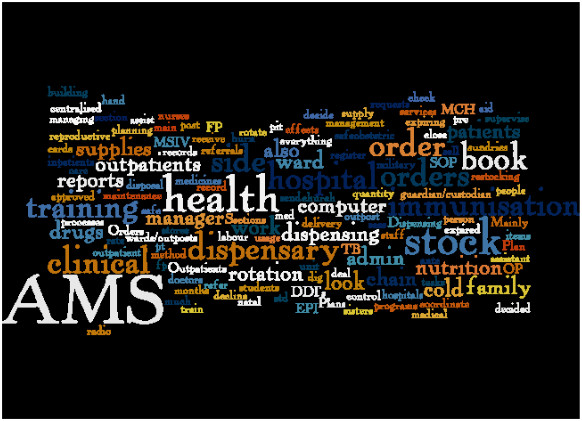
Word cloud generated from Papua New Guinea health personnel responses to the question ‘What are your main responsibilities?’.

Interviewees cited a wide range of day-to-day responsibilities. These are presented in Figure 2 as a word cloud created from the transcribed raw responses of the relevant survey questions. The most frequently reported responsibilities appear in the largest font size. A notable focus is on activities around stock management (e.g. order, report, SOP (standard operating procedures), supplies, stock etc.), and reference to the AMSs, indicating that supply chain activities are recognised as an important aspect of their work.

Six main themes were identified in the data, discussed below.

### Standard operating procedures

We consider that the SOPs for the PNG medical supply chain are generally appropriate. Interviewees articulated a good working knowledge of many of the SOPs required for medical supply management but often actively chose not to follow them in practice. The notable exception to this was a quantification SOP which uses a two-step calculation to determine stock needs. We did not observe any consistent evidence of appropriate understanding of this SOP, or of its use. This had been replaced by estimating or ‘guessing’ as the preferred method of determining orders. Amplifying this issue is the observed practice of excluding the contents of emergency medical supply kits when ordering. These practices lead to the existence of stockouts and wastage, particularly in health centres and provincial hospitals. Wastage of expired medicines and overstocking of near-expired medicines was more common than stockouts.

AMSs and provincial transit store staff play an important role in promoting and training health workers in the SOPs. AMSs managers reported that SOP training was introduced in 2006 through a focused bilateral aid program. SOP training has been implemented with varying degrees of success, with successful implementation seen to rely on the enthusiasm of the individual trainer. It was our observation that SOP trained health personnel had a stronger understanding of, and ability to implement, supply chain practices than those not trained. The main impediment to the success of this program was a slow roll-out of the training due to a lack of funds for training and follow up. One participant reported that in their province only 50% of facilities had health personnel trained in SOPs, and that the ordering system described by the SOPs was relying on consumption data that was more than five years old, preventing appropriate stock levels being maintained. A key issue identified is the role of ongoing supervision of health personnel to ensure that SOPs are implemented at service delivery points. Lack of funds for transport for supervisory visits further amplifies this.

One interviewee had the role of Extended Program of Immunisation (EPI) provincial coordinator. This person was well trained and described a rigorous and methodical supervisory role that they played, including the regular interviewing and observing of staff. This example highlighted the variation in the level of practice of individual personnel and showed what is achievable by suitably motivated and diligent staff.

AMSs are the gateway for medical supplies to primary healthcare facilities. ‘Know-do’ gaps were evident in these facilities and are a significant issue disrupting effective supply. The ability to effectively and transparently ‘screen’ orders to allow equitable distribution of limited supplies against requests was the most significant issue observed. No documented or agreed SOPs were identified to do this, and observation suggests that it is in part based upon AMSs staff personal relationships with facility staff. The outcome is that some facilities routinely receive all of their ordered supplies at the expense of other facilities.

Personnel retention was also cited regularly as a challenge to maintaining best practices around medical supply management, with staff who receive SOP training frequently moving to other facilities, thus losing the medical supply management capacity in the original facility and not implementing their training in the new facility.

### Facilities

A WHO storage conditions checklist [11] was completed for each of the facilities to determine if there were adequate conservation conditions and handling of medicines in the storeroom and dispensing area. The score is the average percentage across all facilities meeting those criteria (n = 15) and is presented in Table 2. It can be seen that there is widespread non-compliance with recommended best-practice, especially within dispensing areas/rooms.

**Table 2:** **Compiled conditions checklist data for 15 Papua New Guinea medical supply facilities**[11]

**Storeroom**	**% True**
1. There is a method in place to control temperature (e.g. roof and ceiling with space between them in hot climates, air conditioners, fans, etc.).	82
2. There are windows that can be opened or there are air vents.	73
3. Direct sunlight cannot enter the area (e.g. window panes are painted or there are curtains/blinds to protect against the sun).	64
4. Area is free from moisture (e.g. leaking ceiling, roof, drains, taps, etc.).	100
5. There is a cold storage in the facility.	73
6. There is a regularly filled temperature chart for the cold storage.	36
7. Medicines are not stored directly on the floor.	55
8. Medicines are stored in a systematic way (e.g. alphabetical, pharmacological).	91
9. Medicines are stored first-expiry-first out (FEFO).	64
10. There is no evidence of pests in the area.	91
11. Tablets/capsules are not manipulated by naked hand.	91
**Average**	**73**
**Dispensing area/room**	
1. There is a method in place to control temperature (e.g. roof and ceiling with space between them in hot climates, air conditioners, fans, etc.).	55
2. There are windows that can be opened or there are air vents.	55
3. Direct sunlight cannot enter the area (e.g. window panes are painted or there are curtains/blinds to protect against the sun).	27
4. Area is free from moisture (e.g. leaking ceiling, roof, drains, taps, etc.).	55
5. There is a cold storage in the facility.	45
6. There is a regularly filled temperature chart for the cold storage.	27
7. Medicines are not stored directly on the floor.	36
8. Medicines are stored in a systematic way (e.g. alphabetical, pharmacological).	55
9. Medicines are stored first-expiry-first out (FEFO).	45
10. There is no evidence of pests in the area.	55
11. Tablets/capsules are not manipulated by naked hand.	45
**Average**	**45**
**Total average**	**60**

Throughout the medical supply chain the physical condition of facilities was variable, with poor structure and layout the predominant observation. In one AMS the main air conditioned medicines storeroom was crowded, with no warehouse racking system and stock crushed under the weight of other stock. Conversely, another AMS had a large amount of underutilised space in the non-air conditioned medical sundries storage area, with a relatively new and well-built warehouse with a large floor area, but with no racking systems. At the time of our visit the air conditioning was not functioning in the adjacent medicines storeroom with fans used in an unsuccessful attempt to control temperatures.

In one provincial hospital many medical supplies were stored haphazardly on the ground in a dilapidated building with broken walls and windows and a leaking roof. This had been the ‘temporary’ storage area for five years since the previous storeroom burnt down. Medicines were mostly stored in a suitable small room within this building, however large amounts of bulk packs of some medicines were observed to be spread amongst the general rubbish on the floor.

### Transport

The ability to transport medical supplies through the supply chain was noted as a significant issue, with the lack of funds (particularly a lack of funding within provinces) for transport amplifying the issues presented by the geography of PNG. With 80% of the population residing in rural areas this issue may be the greatest impediment to effective medical supply [1,6].

Concerns were raised by interviewees about the use of private courier companies to distribute medical supplies from AMSs to health centres. These concerns included the costs associated with the routine use of couriers when health centres cannot organise collection themselves, lack of confirmation of orders arriving at service delivery points, potential for abuse of the system as payment was based on presentation of consignment notes which had been known to be forged, and cold chain failures in non-refrigerated vehicles and when medical supplies were left by couriers at facilities unsupervised.

Other transport issues reported include the use of ambulances for supply activities preventing them being on stand-by for patient transport, the lack of maintenance of grass airstrips at remote health facilities where air is the only transport method, inappropriate storage of medical supplies during transport for long periods on both boats and in vehicles, and lack of funds for transport for supervisory staff visits.

### Emergency medical supply kits

Kits were observed to be underutilised or not used at most service delivery points, and were present in significant excess at some facilities. One remote facility had a very large excess of kits which suggested that it was a repository for kits which were unable to be stored elsewhere in the province. The ongoing ‘push’ distribution of these kits appears to be unnecessary in the provinces visited. Some workers indicated that there was an expectation by supervisors that kits would be distributed to all facilities whether they were needed or not, in support of a political desire to avoid a return to the ‘bad press’ of the previous medical supply crisis.

### The cold chain

Problems with temperature control such as broken air conditioners and fans, broken fridges and airless and hot storage rooms were a consistent feature of the facilities visited, with most facilities exceeding recommended storage temperatures. However; the vaccine cold chain maintained by dedicated EPI staff was observed to be functioning adequately in most facilities, highlighting the opportunity for cold chain compliance with adequate resourcing and training.

The cold chain is often thought to only involve vaccines; however oxytocin and ergometrine maleate used during birth also require refrigeration [14]. Confusing this issue is the use in PNG of brands of oxytocin claiming to meet British Pharmacopeia (BP) standards which are labelled as requiring storage below 30°C.The European Pharmacopeia (which supersedes the BP in signatory European countries) and United States Pharmacopeia standards both require oxytocin and ergometrine maleate to be stored at 2-8°C. There is no internationally recognised pharmaceutical standard which permits these drugs to be stored above these temperatures. Compounding this, some ergometrine maleate used in PNG is labelled as requiring refrigeration; however this was not observed to occur in practice with most supplies found to be stored in unrefrigerated storerooms. These observations call into question the authenticity, quality and efficacy of these products as currently used in PNG. Several interviewees were aware of this issue and expressed concern, but were unable to change the situation as the EPI policy reportedly prohibits the use of the vaccine cold chain for these items.

### Average stockout duration & adequate record keeping

WHO document ‘SF4’ [11] was intended to be used during this research to calculate average stockout duration and assess adequacy of record keeping. This was not possible as no facility had adequate stock records to do this. This indicates that there is a widespread lack of record keeping, supporting the observation and reports that ordering of supplies is done using a ‘best guess’ method rather than calculated from previous or expected consumption. The SOP requiring use of stock cards to record stock levels was not observed to be followed in most facilities, especially health centres.

## Discussion

Potter and Brough [15] provide a model of a systematic approach to achieving sustainable health systems (including medical supply chains) describing the interrelationship between ‘tools’, ‘skills’, ‘staff and infrastructure’, and ‘structures, systems and roles’ in the wider health system. Table 3 shows the results of our research in PNG categorised using the Potter and Brough model.

**Table 3:** **Papua New Guinea medical supply chain issues mapped against the Potter and Brough health system categories**[15]

**Health systems category **[15]	**PNG medical supply chain issues**
**Tools**	● Absence of stock cards and stock records
**Skills**	● SOPs not followed
	● Large variations in worker motivation
	● Supply chain management a low priority
**Staff and infrastructure**	● Facilities in poor physical condition
	● Storage space not managed effectively
	● Temperature control issues
	● Staff retention and movement problems
	● Poor performance of private couriers
**Structures systems and roles**	● Limited budget for SOP training
	● Limited budget for supervisory program
	● Limited budget for transport of medical supplies
	● Use of the cold chain for non-EPI items prohibited
	● Lack of transparency in order screening
	● Suspect quality of oxytocin and ergometrine
	● Use of kits leading to oversupply and wastage

The results of our research supports a clear interrelationship between the four categories: ‘tools’ require ‘skills’, which require ‘staff and infrastructure’, which in turn require ‘structures, systems and roles’. Conversely ‘structures, systems and roles’ enable an effective use of ‘staff and infrastructure’ which enable the use of ‘skills’ which in turn enable an effective use of ‘tools’.

We observed many examples of poor stock management practices, leading to stock-outs and stock excess. There was no apparent pattern to explain the extremes seen, and they appear to be a direct consequence of not following the SOPs. The ‘know-do’ gap between a working knowledge of the systems and procedures of the medicines supply chain, and the actual enacting of them, is a significant issue disrupting effective medical supply. This example indicates that the ‘tools’ are suitable, but that there is a need for review of ‘skills’ and ‘staff and infrastructure’, which also relies on suitable ‘structures, systems and roles’ to be effective.

The cold chain storage of oxytocin and ergometrine maleate is an example of the presence of suitable infrastructure and the absence of a ‘know-do’ gap, but the presence of a specific health program policy which prevents appropriate storage. The intention of this policy is to preserve the integrity of the cold chain to ensure vaccines are stored suitably, but this limitation adversely affects other parts of the supply chain. In this example the need for review of ‘structures, systems and roles’ exists, with support from ‘staff and infrastructure’.

The absence of adequate stock records and stock cards is an example of the need for review of ‘tools’, but is also linked to gaps in ‘skills’, ‘staff and infrastructure’ and ‘structures, systems and roles’ further illustrating the need for a systematic approach to achieving sustainable health systems. Improving the tool (the stock card or record keeping methods) will not solve the problem in the absence of improvements in the other areas.

The absence of regular supervisory visits in rural facilities may leave some heath personnel feeling personally and professionally isolated and is considered to reduce the likelihood of SOPs being followed [16]. As in other PICs, the health personnel who staff these facilities are the backbone of health service delivery, with usually only one or two staff members at each facility. Consistent with our findings in Vanuatu, the clinical workload and responsibilities around the implementation of health programs (e.g. EPI, malaria, maternal and child health, tuberculosis, family planning) mean that medical supply chain management is given a low priority. Suitable care and respect for medical supplies is not routinely observed and potentially reflects a lack of insight into its importance to the successful delivery of healthcare services [1]. Again, different parts of this issue require review of ‘tools’, ‘skills’, ‘staff and infrastructure’ and ‘structures, systems and roles’ to be understood and addressed effectively.

As we have reported previously [1], sustainable health systems strengthening is difficult, especially in resource constrained environments. Similarly to the model of Potter and Brough [15], the inter-related health system building blocks identified by WHO of: service delivery; health workforce; information; medical products, vaccines and technologies; financing, and leadership and governance cannot be considered in isolation [1,17,18]. Our research supports the assertion made by these models that medical supply chain strengthening requires a systematic, inter-related response for sustainable change to occur.

This study provides a snapshot of the issues affecting the medical supply chain in PNG. As in other developing countries, an adequately resourced systematic approach is required to strengthen the medical supply chain. Such a method for improvement is supported by recent WHO approaches and involves consideration of the impact of observations before improvements can be made effectively. [17,18]. The UNFPA-UC research team is using this research to engage governments, pharmacists, doctors, nurses, pharmacy assistants and other pharmacy support workforce cadres to seek a combined solution to identified medical supply competency deficiencies in PICs.

The results of this research have further informed our understanding of the competencies required by health personnel to conduct medical supply management activities effectively in PICs. It is our endeavour to further explore these competencies and to develop training approaches that will meet local requirements for competency development so that further progress toward reaching the MDGs can be achieved.

## Limitations

Time, geography and funding influenced the number and location of health facilities visited. Although we visited some quite remote facilities, we did not experience first-hand the supply issues facing extremely remote facilities which are common in PNG. We also only visited 3 of the 20 areas in PNG, potentially limiting the applicability of our findings to all of PNG. These important limitations to our results are partially mitigated by the discussion of remote supply issues and country-wide issues with a wide range of interviewees and within the focus groups.

## Conclusion

This research provides further evidence that the factors influencing the availability of medical supplies in PNG consist of a range of interrelating issues that can be classified under: ‘tools’; ‘skills’; ‘workers and infrastructure’; and ‘structures, systems and roles’. These issues consist of both simple and complex problems involving the different levels and cadres of workers within the medical supply chain. Health systems sustainability theory suggests that a coordinated approach which addresses the inter-related nature of these issues, led by the PNG government and supported by suitable development partners, will be required for sustainable health systems change to occur. These changes are necessary for PNG to meet the health-related Millennium Development Goals.

## Competing interests

The authors declare that they have no competing interests.

## Authors’ contributions

AB was responsible for the original concept and design of the manuscript. AB and BG collected and analysed the data and drafted, revised and approved the manuscript.
